# Patterns of between-farm contacts via professionals in Sweden

**DOI:** 10.1186/s13028-014-0070-2

**Published:** 2014-11-04

**Authors:** Emelie Olofsson, Maria Nöremark, Susanna Sternberg Lewerin

**Affiliations:** Department of Biomedical Sciences and Veterinary Public Health, SLU, Swedish University of Agricultural Sciences, Box 7036, Uppsala, SE-750 07 Sweden; Department of Disease Control and Epidemiology, SVA, National Veterinary Institute, Uppsala, SE-751 89 Sweden

**Keywords:** Contact data, Contact frequency, Farm visitors, Geographic pattern, Biosecurity

## Abstract

**Background:**

Infectious diseases of livestock have negative consequences for animal production as well as animal health and welfare and can be transmitted between farms via direct (live animal movements) as well as indirect (via physical vectors such as, people, transport vehicles and fomites) contacts. The objective of the study was to examine the travel patterns of professionals visiting Swedish farms (veterinarians, milk tanker drivers, artificial inseminators, maintenance technicians and livestock hauliers). This was done by obtaining records of the farms visited by a sample of professionals in the above categories in one week in January, one week in April, one week in July and one week in October in the Swedish counties Västerbotten, Södermanland, Västergötland and Skåne.

**Results:**

There were twelve participating organisations, and data was provided for one to three individuals/vehicles/veterinary practices per professional category and per geographic region (except for dairy service technicians and livestock hauliers who did not provide data from all regions). There was a trend towards larger areas covered and smaller number of farms visited per week in the north, but exceptions occurred and there were regional variations. Generally, the greatest areas were travelled by milk tankers and livestock hauliers, and the profession travelling over the smallest areas tended to be the veterinarians. Milk tankers visited most farms per week, one milk tanker could visit between 23 and 90 farms per week and travel over areas between 717 km^2^ and 23,512 km^2^ per week.

**Conclusions:**

Valuable insight into the travel patterns of Swedish professionals has emerged although the implications of the study largely concern highly infectious diseases. Movement of live animals pose the greatest risk for the spread of infectious animal diseases; however indirect contacts are important for many diseases. The results of this study indicate that in Sweden a highly contagious disease might spread over a large area in the time span of one incubation period, which ought to be kept in mind in case of an outbreak and in outbreak investigations. The difficulties in contacting some professionals visiting farms could be a problem in an outbreak situation.

## Background

Infectious diseases of livestock have consequences for animal production as well as animal health and welfare. The transboundary animal diseases (TAD:s) are also a concern for international trade [1,2] and outbreaks of such diseases may cause enormous losses for society as a whole [3]. For the individual farmer, endemic diseases are usually of more interest, as these diseases have a higher impact on everyday production. Transmission between herds occurs via direct (live animal movements) as well as indirect (via physical vectors such as, people, transport vehicles and fomites) contacts [4]. Transmission via live animals represents the highest risk as moving an infected animal also moves the infection, but other routes of transmission are also important, as has been seen in various disease outbreaks [5,6]. The risk posed by each contact between farms depends on disease prevalence and the biosecurity measures taken to reduce the risk. There is spatial variation in many risk factors for endemic diseases. The density of the susceptible species can also be reflected in the geographical distribution of a specific disease [7,8] and a high animal density is often regarded as a risk factor for contagious diseases. There are also other geographical influences, as is seen in for example the prevalence of *Coxiella burnettii* [9] and verotoxin producing *Escherichia coli* in Swedish cattle herds [9] and *Salmonella* Dublin in Denmark, United Kingdom and Sweden [9-11]. The geographical risk reflects various transmission routes that occur more frequently in the local environment [12]. This is taken into account in the restriction zones laid down around infected premises in most disease control plans for TAD:s, such as e.g. African swine fever and foot-and-mouth disease [13,14]. Insight into the geographical patterns of between-farm contacts is therefore important for exotic disease control. Some contact patterns can be associated with the prevalence of endemic diseases and may be used for assessing the risk for individual farms as regards endemic diseases [15,16]. Biosecurity measures are essential for risk reduction and the application of such measures has been studied in various European countries [17-19], including Sweden [20,21]. The registration of livestock movements is mandatory in the European Union [22-24] and patterns of such movements have been studied in different EU member states e.g. UK and Sweden [25,26]. Other contact patterns between farms are however less studied as such movements are rarely registered centrally, but the frequency of various contacts on farm level has been studied [27,28]. To our knowledge, a geographical representation of actual indirect contacts between Swedish animal holdings has not been demonstrated. For the Swedish situation, assessing the geographical patterns of indirect contacts between farms has been one of the missing pieces of information in order to gain insight into the overall contact pattern between livestock holdings and the risk presented by these contacts. The aim of this study was to fill that knowledge gap.

## Methods

### Study population

In 2012, there were 19,561 cattle holdings, 9,263 sheep holdings, and 1,318 pig holdings in Sweden, mainly located in the southern parts of the country [29]. The average herd sizes were: 70 dairy cows, 17 beef cows, 186 sows, 765 fattening pigs and 32 adult sheep. The highest density of cattle is in the southwest of the country, where the counties Västra Götaland and Skåne have approximately 0.15 holdings/km^2^ and 0.22 holdings/km^2^, respectively. The lowest density of cattle is found in the northern third of the country, where Norrbotten in the far north has the lowest density (0.002 holdings/km^2^) and Västerbotten has the highest density (0.01 holdings/km^2^) of the northern counties. The county of Södermanland lies somewhere in between with a cattle density of 0.09 holdings/km^2^ and a pig density of 0.01 holdings/km^2^). The highest density of pig holdings can be found in Skåne (0.06/km^2^), while Västra Götaland has 0.016/km^2^ and Västerbotten only 0.001/km^2^. Sheep are more evenly distributed but most holdings are found in the southern half of the country. The density of sheep holdings is around 0.06/km^2^ in Västra Götaland, Skåne and Södermanland, but only 0.004/km^2^ in Västerbotten.

### Data collection

Six categories of organisations with staff that visit farms in their daily work were initially contacted via e-mail or phone. The organisations contacted were the District Veterinary Organisation in the Swedish Board of Agriculture, three regional dairy companies, 41 regional livestock hauling companies, two organisations providing artificial insemination services for cattle famers (one national, one regional), the major producer of dairy machinery and the major pest control company. The aim of the study was explained in the e-mail or during the initial phone call and the organisation was asked to participate. The participants were ensured anonymity for themselves and their customers. If originally contacted by e-mail, this was in some cases followed up by a phone call.

**Figure 1:**
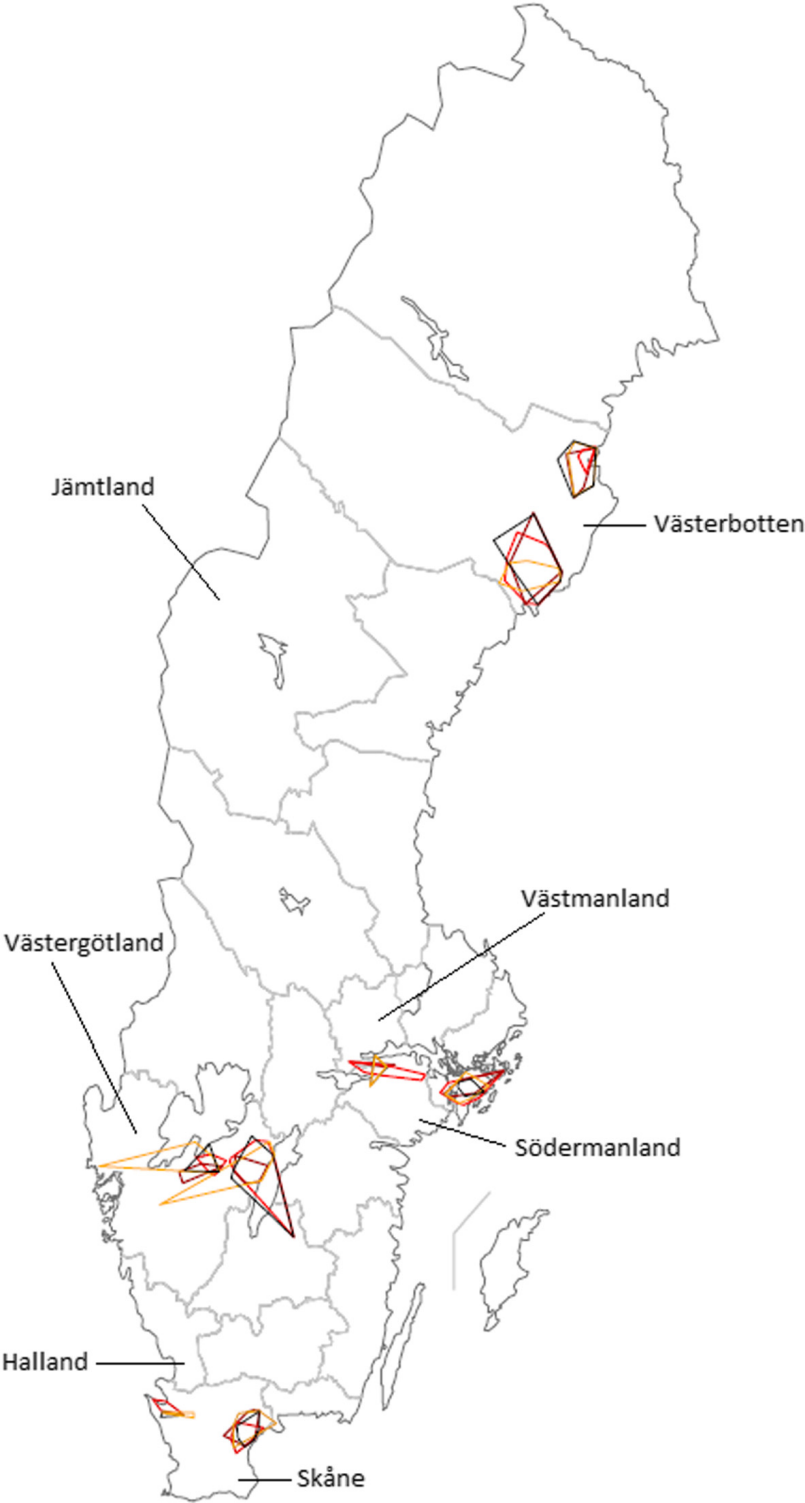
**Geographic area travelled by Swedish veterinarians, per veterinary practice and week.** Travel area of Swedish veterinarians employed at eight mixed practices in 2012 and 2013 as reported in Vet@Journal, scale 1: 7 500 000. Time periods depicted: 8 – 14 October, 2012: yellow; 7 –13 January, 2013: black; 8 – 14 April, 2013: red; 8 – 14 July, 2013: brown.

The organisations choosing to participate were asked to provide either the addresses or the unique identities of the holdings visited by at least one employee (or, in the case of the dairy companies, at least one milk tanker and one driver) on each day during the following periods: 8–14 October 2012, 7–13 January 2013, 8–14 April 2013 and 8–14 July 2013. These periods were chosen to be as representative as possible of all four agricultural seasons in all selected counties. If the participants were unable to provide this information, other alternatives discussed included postal addresses or number of farms visited and distance driven each day. The geographic areas chosen were Västerbotten (in the north of Sweden), Södermanland (in the southeast of Sweden), Västergötland (in the southwest of Sweden) and Skåne (in the very south of Sweden), see Figure 1. The geographical regions were chosen to represent different regions with different livestock population structure and because previous studies with a similar focus were carried out in these regions [20,30]. The farms of interest were farms with cattle, pigs, sheep and goats.

The data were collected via e-mail or ordinary mail, with the exception of the data from the veterinary organisation that were collected from a central database of veterinary medical records (Vet@Journal). The medical records that included the unique identity of the holding (required when treating livestock) were examined and, if it was evident a farm had been visited by a veterinarian, that unique identity was noted. Treatments of pets were included if this had taken place on a farm with livestock. For farm visits related to sampling for food chain information, the billing date was used as the date of the visit (as no regular medical records were kept of those visits). Visits by all veterinarians of the chosen veterinary practices during the chosen dates were registered.

### Data management and editing

The unique identity of the holdings were converted into coordinates or, in case a coordinate was missing, a postal address. The coordinates/postal addresses were extracted from the animal holding database of the Swedish Board of Agriculture. The postal addresses or postal codes were converted into coordinates using internet based search services. A small number of holdings shared the same postal code and had no other identification. These holdings were assigned the same coordinates. Two different coordinate systems (World Geodetic System 1984 and Rikets triangelnät 1990) were used, due to the Swedish Board of Agriculture using a different geographical system for their recording than what was used to originally convert the addresses provided by the participants into coordinates.

### Data analyses, software used

ArcMap (GIS, ArcView, ESRI, Redlands, CA, USA) was used to plot the coordinates on to a map of Sweden. As the individual farm locations were not relevant for the study, polygons were used to illustrate the areas travelled by the visitor categories.

The areas travelled per day and per week were calculated using ArcMap, and the descriptive statistics were calculated using Microsoft Excel® 2007 (Microsoft Co., Redmond, USA). Descriptive statistics included mean, median and range for area travelled and number of farms visited, as well as ratio of area/number of farms visited. If the same farm was re-visited during the same period of interest it was only included once in the area calculations as well as in the number of farms visited and subsequent statistical analyses.

## Results

### Response rate

Out of 49 contacted organisations, 24 (49%) could not be reached by phone or e-mail (all of these were livestock hauliers) and out of the 25 that were reached, twelve chose to participate (48%). Only five of the livestock hauliers chose to participate (29% of livestock hauliers that were reached), while among the other organisations only the pest control company chose not to participate. The company with dairy maintenance technicians was unable to provide data from all regions of interest.

The reasons given for not participating included: time-consuming (33%, n =4), too many farms visited (8%, n =1) and no longer in the business (8%, n =1). In addition, 50% (n =6) gave no reason for not participating.

### Descriptive statistics

**Figure 2:**
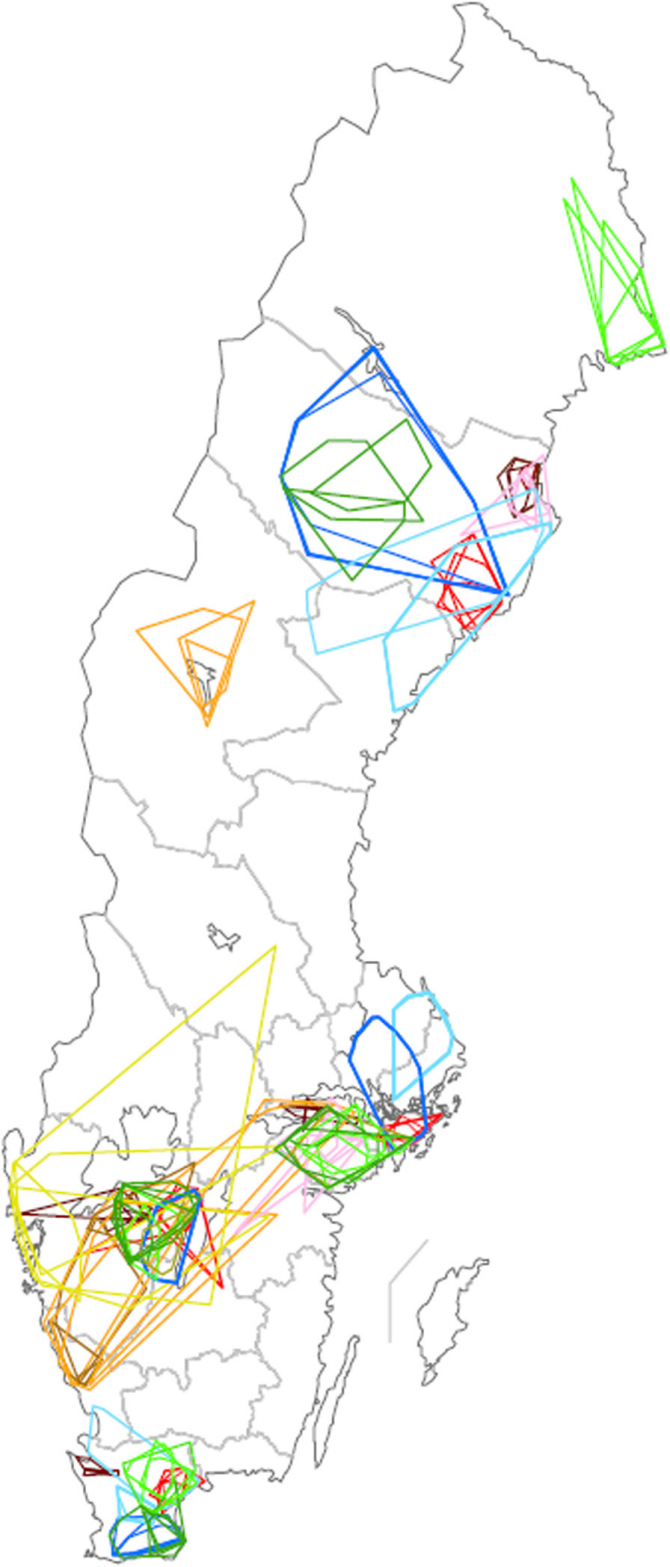
**Geographic area travelled by Swedish veterinarians, milk tankers, artificial inseminators, dairy service technicians and livestock hauliers, per week.** Travel area of veterinarians employed at eight veterinary practices, seven milk tankers, ten artificial inseminators, two: dairy service technicians and four Swedish animal transports in 2012 and 2013, scale 1: 7 500 000. Time periods depicted: 8 – 14 October 2012 or 7 – 13 October 2013, 7 –13 January 2013, 8 – 14 April 2013 or 7 – 13 April 2013 and 8 – 14 July, 2013. Red and brow: veterinarians, blue: milk tankers, green: artificial inseminators, pink: dairy service technicians, orange and yellow: livestock hauliers*.* Individuals/vehicles/veterinary practices separated by hue.

All descriptive statistics are summarised in Table 1. No seasonal pattern could be seen in the data and thus all time periods were analysed together. The areas covered by all different categories of farm visitors are illustrated in Figure 2.

**Table 1:** **Area travelled and number of farms visited per week by geographic region**

		**Västerbotten**			**Södermanland**			**Västergötland**			**Skåne**			**Jämtland**			**Västmanland**			**Halland**		
		**Mean**	**Median**	**Range**	**Mean**	**Median**	**Range**	**Mean**	**Median**	**Range**	**Mean**	**Median**	**Range**	**Mean**	**Median**	**Range**	**Mean**	**Median**	**Range**	**Mean**	**Median**	**Range**
Veterinarians																						
	Area (km^2^)	1637	1376	220-3208	457	415	65-1021	1366	1234	318-2666	426^1^	486^1^	59-898^1^	-	-	-	-	-	-	-	-	-
	Number of farms	14	12	9-23	6	6	3-11	24	17	7-55	9	8	1-17	-	-	-	-	-	-	-	-	-
	Ratio, area/number of farms	117			76			57			47			-	-	-	-	-	-	-	-	-
Milk tankers																						
	Area (km^2^)	17842	19585	11518-23512	3674	2170	2170-5679	3066	3066	NA	1683	1101	717-5074	-	-	-	-	-	-	-	-	-
	Number of farms	45	44	30-66	61	63	57-64	89	89	88-90	48	47	23-84	-	-	-	-	-	-	-	-	-
	Ratio, area/number of farms	396			60			34			35			-	-	-	-	-	-	-	-	-
Artificial inseminators																						
	Area (km^2^)	5001	5307	2356-6874	2892	3013	1044-4661	2587	2521	1495-4018	1660	1481	745-2821	-	-	-	-	-	-	-	-	-
	Number of farms	13	12	7-18	20	20	14-28	38	39	22-49	21	21	11-29	-	-	-	-	-	-	-	-	-
	Ratio, area/number of farms	385			145			66			79			-	-	-	-	-	-	-	-	-
Dairy service technicians																						
	Area (km^2^)	1116	1233	63-1936	1730	1679	1235-2327	-	-	-	-	-	-	-	-	-	-	-	-	-	-	-
	Number of farms	4	5	2-5	6	7	4-8	-	-	-	-	-	-	-	-	-	-	-	-	-	-	-
	Ratio, area/number of farms	279			288			-	-	-	-	-	-	-	-	-	-	-	-	-	-	-
Livestock hauliers																						
	Area (km^2^)	-	-	-	-	-	-	21331	16457	4275-48134	NA	NA	NA	3568	3621	1567-5517	NA	NA	NA	9748	11925	155-18715
	Number of farms	-	-	-	-	-	-	20	19	18-22	NA	NA	NA	11	11	9-12	1	1	NA	12	15	4-18
	Ratio, area/number of farms	-	-	-	-	-	-	1067			NA	NA	NA	324			NA	NA	NA	812		

NA = not applicable due to data format.

^1^ = one value not included in statistical analyses due to data format.

- = data missing.

The area travelled and number of farms visited per week by Swedish veterinarians, milk tankers, artificial inseminators, dairy service technicians and livestock hauliers.

### Veterinarians

Eight veterinary practices with large animal practice were chosen for the study, two in Västerbotten, two in Södermanland, two in Västergötland and two in Skåne. In each veterinary practice, between three and ten veterinarians were employed during the time periods of interest.

The largest mean area travelled by veterinarians per week was found in Västerbotten, both in terms of the largest mean area travelled by all working veterinarians in a veterinary practice in a week (1,637 km^2^, range 220–3,208 km^2^) and in terms of the mean area travelled by a single veterinarian in a week (507 km^2^, range 5–2,010 km^2^). The smallest area travelled per week per veterinary practice could be found in Skåne (426 km^2^, range 59–898 km^2^). However, in Södermanland, a single veterinarian on average travelled over the smallest area per week (144 km^2^, range 1–446 km^2^). The area where the highest number of farms per veterinary practice and individual veterinarian was visited per week was Västergötland (24, range 7–55 and 7, range 2–27 respectively) and the region where the lowest number of individual farms per veterinary practice and individual veterinarian was visited per week was Södermanland (6, range 3–11 and 3, range 2–6 respectively).

The largest mean area travelled by a veterinary practice in a day could be found in Västerbotten (390 km^2^). The smallest area travelled per day per veterinary practice could be found in Skåne (120 km^2^).

For further data, see Table 1. For a visual representation of the area travelled by each veterinary practice per week, see Figure 1.

### Milk tankers

Data from two milk tankers in Västerbotten, two in Södermanland, one in Västergötland and two in Skåne were obtained.

**Figure 3:**
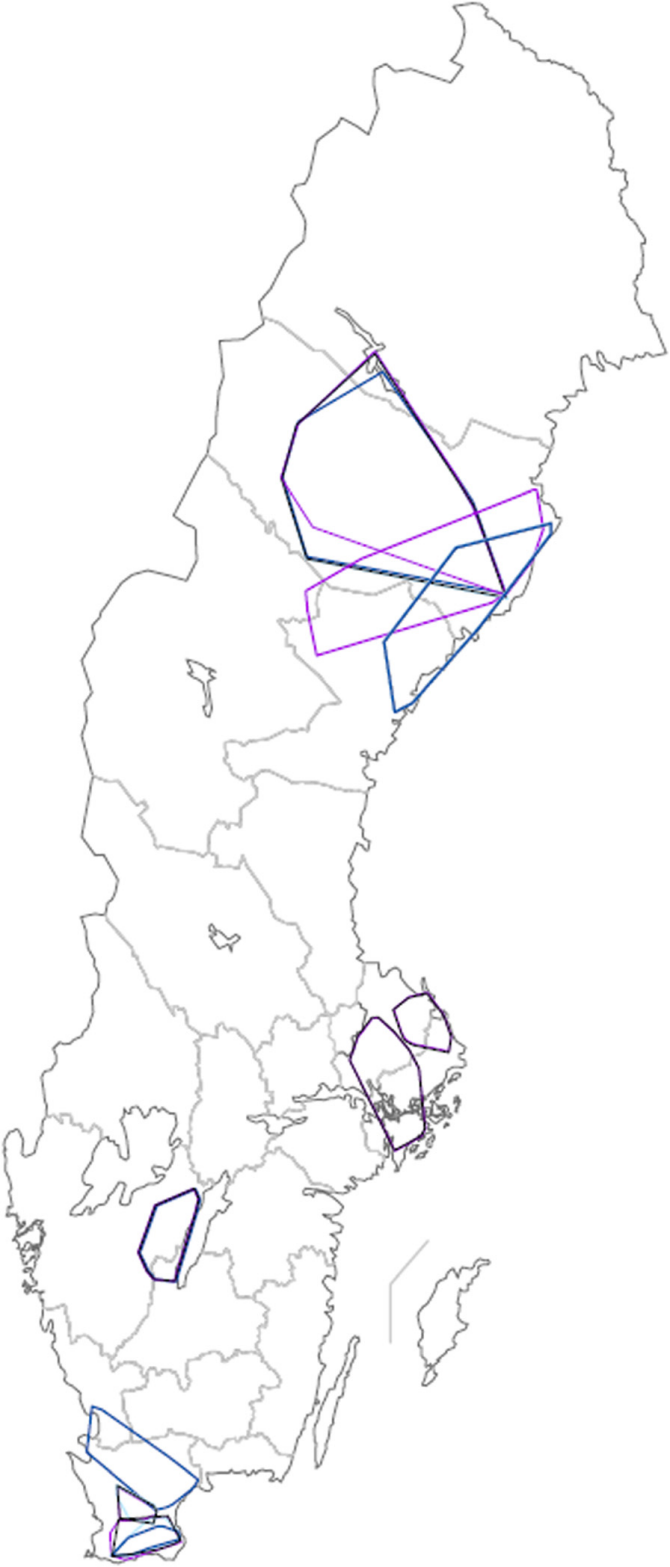
**Geographic area travelled by Swedish milk tankers, per week.** Travel area of seven Swedish milk tankers from three different dairy companies in 2012 and 2013, scale 1: 7 500 000. Time periods depicted: 8 – 14 October, 2012 or 7 – 13 October 2013: purple; 7 –13 January, 2013: black; 8 – 14 April 2013: light blue; 8 – 14 July: dark blue.

The largest mean area travelled by a milk tanker per week could be found in Västerbotten (17,842 km^2^, range 11,518-23,512 km^2^) and the smallest mean area travelled per week could be found in Skåne (1,683 km^2^, range 717–5,074 km^2^). The area with the highest number of individual farms visited per milk tanker per week was Västergötland (89, range 88–90) and the region with the lowest number of individual farms visited per milk tanker per week was Västerbotten (45, range 30–66). For further data, see Table 1. The geographic areas covered by the milk tankers are shown in Figure 3.

### Artificial inseminators

Data from two artificial inseminators in Västerbotten, three in Södermanland, three in Västergötland and two in Skåne were obtained.

The largest mean area travelled by an individual artificial inseminator per week could be found in Västerbotten (5,001 km^2^, range 2,356-6,874 km^2^) and the smallest mean area travelled per week could be found in Skåne (1,660 km^2^, range 745–2,821 km^2^). The highest number of individual farms per artificial inseminator visited per week was in Västergötland (38, range 22–49) and the region where the lowest number of individual farms per artificial inseminator was visited per week was Västerbotten (13, range 7–18). For further data, see Table 1.

The largest mean area travelled by an individual artificial inseminator per day could be found in Västerbotten (757 km^2^, range 57–2,302 km^2^) and the smallest mean area travelled per day could be found in Västergötland (404 km^2^, range 36–1,403 km^2^).

**Figure 4:**
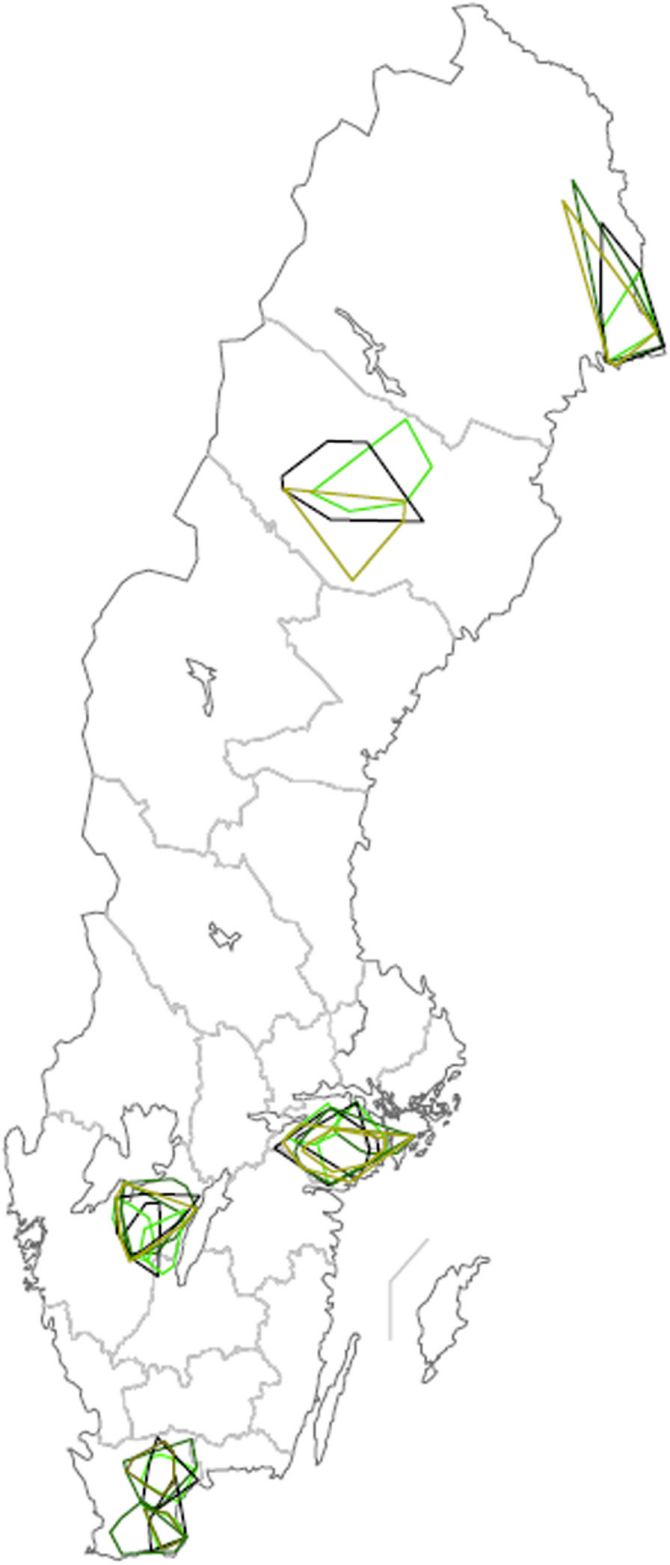
**Geographic area travelled by Swedish artificial inseminators, per week.** Travel area of ten Swedish artificial inseminators employed by two different companies in 2012 and 2013, scale 1: 7 500 000. Time periods depicted: 8 – 14 October, 2012 or 8 – 14 November, 2012: olive green; 7 –13 January, 2013: black; 8 – 14 April: light green; 8 – 14 July, 2013: dark green.

The areas travelled by each artificial inseminator per week are illustrated in Figure 4.

### Dairy service technicians

Data from one dairy service technician in Västerbotten and one in Södermanland were obtained. One could not provide data for October 2012 but provided data for the corresponding week in 2013.

In Södermanland, the mean area travelled by an individual technician per day was 147 km^2^ (range 49–322 km^2^) and two farms were visited per technician per day (range 1–3). In Västerbotten, the mean area travelled by an individual technician per day was 288 km^2^ (range 63–526 km^2^) and one individual farm per technician per day was visited.

**Figure 5:**
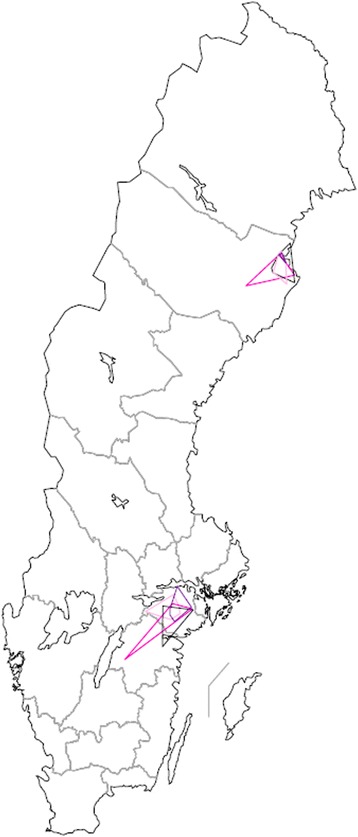
**Geographic area travelled by Swedish dairy service technicians, per week.** Travel area of two Swedish dairy service technicians employed by one company in 2012 and 2013, scale 1: 7 500 000. Time periods depicted: 8 – 14 October, 2012: dark purple; 7 –13 January, 2013: black; 8 – 14 April, 2013 or 15 – 21 April, 2013: light pink; 8 – 14 July, 2013: dark pink.

There were no major differences in mean area travelled and mean number of farms visited per week between service technicians in Södermanland and in Västerbotten. For further data, see Table 1. The areas travelled by each service technician per week are illustrated in Figure 5.

### Livestock hauliers

Data from one livestock haulier in Västergötland, one in Skåne, one in Jämtland, one in Västmanland and one in Halland were obtained. The hauliers in Skåne and Västmanland were excluded from area calculations due to data limitations as explained below.

Out of the data provided the livestock haulier in Västergötland travelled, on average, over the largest area per week (21,331 km^2^, range 4,275-48,134 km^2^) and the livestock haulier in Jämtland (sharing borders with Västerbotten) travelled, on average, over the smallest area per week (3,568 km^2^, range 1,567-5,517 km^2^). The livestock haulier in Västergötland visited the largest mean number of farms per week (20, range 18–22).

The livestock haulier in Västmanland travelled on average 19 km, range 2–30 km, visiting one farm each week. For the livestock haulier in Skåne, only distance in kilometres travelled each day was specified. The maximum distance travelled per week was in July 2013 (742 km) and the minimum was in January 2013 (430 km).

Among hauliers for which the area could be calculated, the livestock haulier in Västergötland travelled over the largest mean area per day (3,361 km^2^, range 88–33,452 km^2^) and the livestock haulier in Halland over the smallest (703 km^2^, range 19–3,293 km^2^). The livestock hauliers in Skåne and Västergötland visited the largest mean number of farms per day (5, range 1–8).

**Figure 6:**
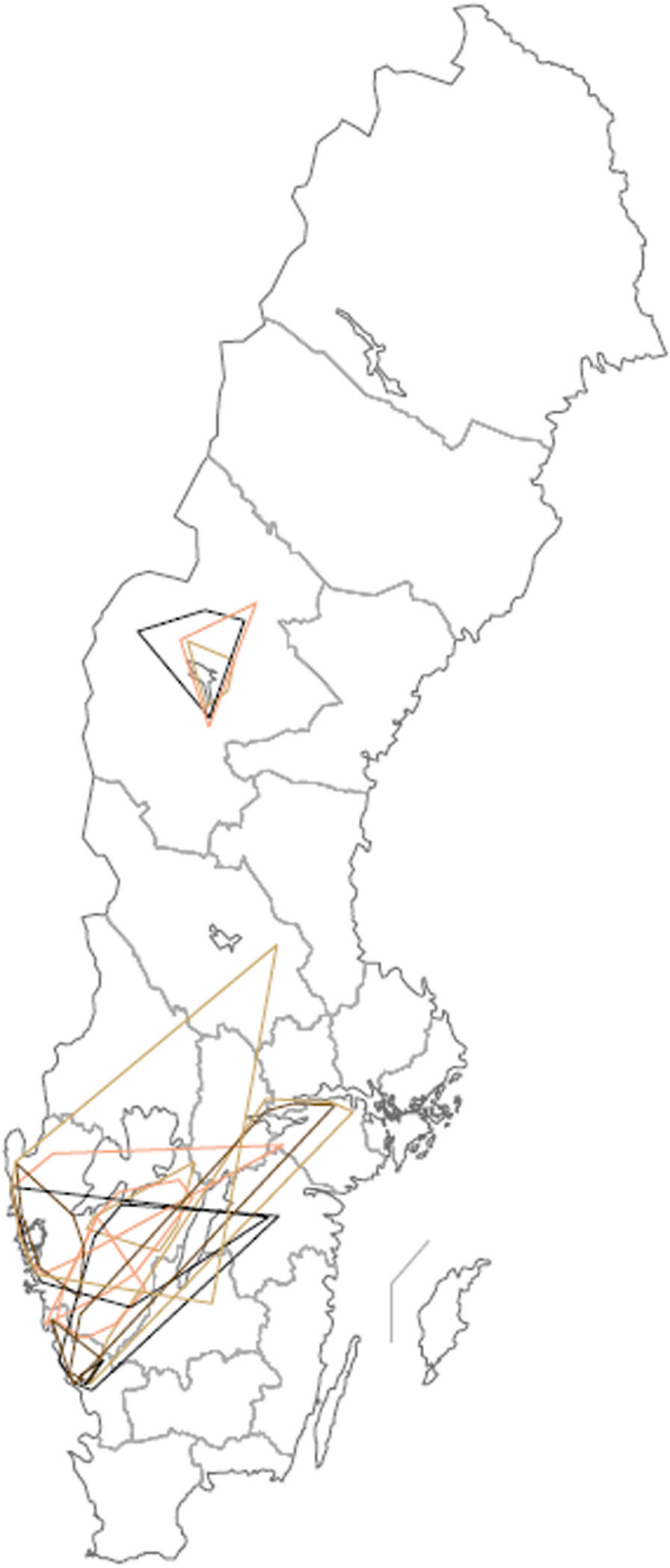
**Geographic area travelled by Swedish livestock hauliers, per week.** Travel area of four Swedish animal transports, operated by three companies transporting animals professionally in 2012 and 2013, scale 1: 7 500 000. Time periods depicted: 8 – 14 October 2012 or 7 – 13 October 2013: orange, 7 –13 January, 2013: black, 8 – 14 April: light brown, 8 – 14 July, 2013: dark brown.

For further data, see Table 1. The area covered by each livestock hauiler (vehicle) per week is illustrated in Figure 6. The livestock hauliers in Skåne and Västmanland were excluded from Figure 6 due to their data format.

## Discussion

This study was limited by data constraints and thus general inference cannot be drawn from the results. Based on experience from previous studies where difficulties in obtaining contact information and data from some groups of professionals were encountered [21], it was decided to focus on a limited number of groups. An all-encompassing study including all relevant areas and professions was regarded as unfeasible. Thus, some important indirect transmission routes, e.g. claw trimmers, are missing from the study, although they constitute a major risk of between-farm spread of diseases. Another group that was excluded was deadstock collectors, but here the reason was that they seldom enter the farm [20].

The time periods were chosen to be representative of all seasons and to avoid major holidays, as studies have demonstrated a deviation in e.g. recorded animal movements around major holidays such as Christmas [31,32]. The geographical areas were chosen to be representative of different livestock densities and animal production types.

Although the results indicate large heterogeneities, these were not unexpected and are most likely not entirely attributable to the limited sample. As discussed below, there are regional variations due to differences in animal population densities. Moreover, the work of many of the professional categories is event-driven, i.e. they cannot plan their visits on a weekly basis as they are called out to farms on short notice, when they are needed. Thus, despite the small sample size (both as regards number of professional categories and number of respondents within each category) we believe that the results reflect at least part of the potential for geographic spread of livestock diseases between Swedish livestock farms.

### Veterinarians

The veterinary organisation chosen is nationwide and governmental. All veterinary practices providing data are mixed practices, i.e. covering livestock as well as pets and horses, but the proportions differ between the veterinary practices. Clients seeking treatment for their pets will most often visit the veterinary clinic, while the veterinarians visit the farms for treatment of livestock. Some veterinary practices will treat horses on their premises, while in others the veterinarians visit the stable. Because of the difficulties ascertaining whether or not a farm had been visited for horse treatments, and horses being outside the focus of the study, all records of horse treatments were excluded.

The proportion of livestock, small animals and horses might be reflected in the number of farms visited by veterinarians from each veterinary practice. While the number of farms visited by a veterinary practice in a week in Skåne can be as few as one (see Table 1), this may be due to a high proportion of small animal patients and/or horses. As each farm was only counted once, repeat visits to the same farm in the same week are not reflected in the results. Furthermore, competing private veterinary practices may operate in the same area as the government organisation and their numbers vary in different parts of Sweden. The total number of private large animal practitioners is smaller than the number of large animal practitioners within the government organisation and most of the private practitioners work in the southern parts of the country, but the proportion of livestock covered by one or the other is not known.

Veterinarians do typically not plan their visits to farms days ahead, the pattern of their travel will generally be governed by chance and therefore difficult to predict. However, the data collected over time should give a realistic view of their travelling patterns.

Much of the analyses done on the data obtained for the veterinarians were done on the level of the veterinary practice, treating all veterinarians working in a particular week as a unit. This was done to avoid the skewedness in daily data but also based on the fact that veterinarians in the same veterinary practice share vehicles, equipment and facilities and the risk of cross-contamination in the case of the extremely infectious animal diseases (e.g. foot-and-mouth disease, classical or African swine fever) is deemed sufficiently high so as to regard all veterinarians in the same practice as one unit. Biosecurity routines that will probably be sufficient for less contagious diseases may not be enough in case of an outbreak of a highly contagious disease.

The large area of travel in Västerbotten was not unexpected, as farm density in the north of Sweden is much lower than in the rest of the country. The mean area travelled by the veterinarians in Västergötland was also quite large (see Table 1). However, the veterinarians in Västergötland visit many more farms in a week, most likely explaining the large area compared to e.g. Södermanland and Skåne.

### Milk tankers, AI technicians and dairy service technicians

Three large dairy companies provided the data for the milk tankers and data from the same seven vehicles were obtained for all time periods. However, the drivers varied (both over the year and over the day, as milk tanker drivers tend to work in shifts) and the same vehicle could be running different routes. Some dairy companies also provided data on individual milk tanker drivers. From this data it was clear that drivers may be on different routes and therefore visiting farms in a much larger area. Data provided from Västerbotten exemplified this, where one driver travelled in one week in January 2013 over an area of 25,112 km^2^. This is 1,600 km^2^ larger than the greatest area travelled by any single vehicle in the study.

Because milk tankers follow planned routes there were only small deviations in the patterns. One exception was seen in Skåne in July (see Figure 3), where one of the tankers broke its pattern. This might be due to vacations and temporary employees over the summer.

The same pattern as for veterinarians, with the largest area covered in Västerbotten and the smallest in Skåne, was seen for the milk tankers. Similarly, the ratio between the average area travelled and number of farm visited per week was much higher in Västerbotten than in Västergötland, reflecting dairy herd density. As expected, the movement patterns of AI technicians were similar as they also move between dairy herds.

Dairy service technicians maintaining milking systems would be expected to have similar movement patterns, but only two service technicians were able to participate in the study. There were no great differences in area travelled per week or number of farms visited between the service technicians in Västerbotten and Södermanland, but due to limited data no conclusions can be drawn from this.

### Livestock hauliers

All 42 hauliers listed by the Board of Agriculture as licensed to transport livestock in Västerbotten, Södermanland, Västergötland, Skåne and neighbouring counties were contacted. Only five provided data on one or more vehicles. Out of the four selected geographical areas, participants in this category could only be found in Västergötland and in Skåne, and the data obtained was limited.

Livestock hauliers are a heterogenous group, ranging from full-time professionals with several vehicles and a number of employees to small abattoirs that transport animals sporadically. The heterogeneous nature of this group is reflected in their travel pattern. Earlier studies have shown that Swedish animals may be transported up to 1,200 km [32]. In contrast to the other groups in this study, drivers do not always return to a point of origin each day but may be required to sleep in or near their vehicle. Thus, they may travel long distances and over large areas in a week, as seen in Figures 6 and 2. While some vehicles in this study visited a very small number of farms in a week and travelled over weekly distances as short as 2 km, others could cover weekly areas as large as 48,134 km^2^. Even in a 24 h time span, a single vehicle in Västergötland travelled over an area of 33,452 km^2^. The weekly area range of the same vehicle could vary between 4,275 km^2^ and 48,134 km^2^, making livestock hauliers a profession whose movement patterns are very difficult to predict.

The largest mean area travelled by a vehicle in a week was in Västergötland, breaking the pattern of the veterinarians, milk tankers and artificial inseminators. However, although data on one vehicle per day was provided, it was unclear whether or not it was the same vehicle for all days in the period.

### General implications

Despite the limited data valuable insight into the travel patterns of Swedish professionals has emerged. While milk tankers can mostly be relied upon to visit a set number of farms, livestock hauliers cannot. The trend seems to be that larger areas and a smaller number of farms are visited per week and per day in the north. However, exceptions occur, which is important to keep in mind. There were also clear variations within the regions, especially as regards veterinarians. Generally, the professions travelling across the greatest areas were milk tanker drivers and livestock hauliers, and the profession travelling over the smallest areas was, as a rule, the veterinarians. Although general conclusions about movement patterns of different professional categories are hampered by the limited data, the study clearly demonstrates that individual movements outside the general patterns may occur. Some of the heterogeneities might have been reduced in a larger and more comprehensive dataset, allowing for a better overall picture. However, the observed variations are of interest and indicate the need for detailed contact tracing in all disease outbreaks.

High farm density has been identified as a risk factor regarding the transmission of infectious diseases [7,8] and regions with a high number of farms and high degree of indirect and direct contacts are potential risk areas for the spread of infectious animal diseases. While professionals in the north of Sweden seem to travel across greater areas, in general a smaller number of farms are visited. A disease might therefore not spread as rapidly as in some of the more densely populated areas of the country, although there are other factors affecting this risk. The number of cattle and pigs transported into the north of Sweden is lower, compared to other regions [32,33] and a disease may therefore be less likely to enter the region.

Certain professionals may visit a large number of farms in one week (mainly milk tankers and AI technicians), and can thus spread a disease rapidly. Although the movement of live animals pose the greatest risk for the spread of infectious animal diseases, indirect contacts are important for many diseases [4-6] and it stands to reason that great care should be taken to minimise the risks of indirect transmission as well. The biosecurity of both farms and professional visitors play an important part in the protection against infectious animal diseases [4-6]. Biosecurity routines vary among professionals as well as between farms [18-20] and many professionals visiting Swedish farms have reported difficulties in maintaining an adequate level of biosecurity [21].

The implications of the study largely concern the highly infectious diseases, such as foot-and-mouth disease, classical and African swine fever. In case of an outbreak of an epizootic disease, a restriction zone is laid down around the infected holding. This restriction zone has a radius of at least 10 km, i.e. an area of approximately 314 km^2^. The results of this study indicate that, in Sweden, a highly contagious disease might have spread over a larger area in the time span of one incubation period. This ought to be kept in mind in case of an outbreak and in outbreak investigations.

### Records

The most easily obtained data were on the milk tankers. The dairy companies use computer registers and well planned routes. The participating veterinary organisation also keeps electronic records which made the data readily available. Unfortunately, as veterinary records are not kept with disease traceability foremost in mind, sorting out the relevant information was highly time-consuming and some information may have been lost due to incomplete or unclear records. Even though there were difficulties in getting data on dairy service technicians from all geographical regions of interest, the two contributing technicians provided their data in a timely manner, likely due to the limited number of farms visited. The organisations providing artificial insemination most likely keep electronic records of their visits and were able to provide the data needed.

Although livestock movements in the European Union must be registered [22-24], the official registers do usually not contain information on travel routes. Such data must be obtained from the hauliers and are not always easily accessed. Out of the livestock hauliers choosing not to participate, reasons such as “too many farms”, “very time-demanding” and “too busy at the moment” were given. Two of the participating companies did not have email accounts and some datasets were hand-written, indicating that there may be a lack of electronic records.

The difficulties obtaining data, especially from the livestock hauliers, could be seen as worrying, however one can expect that in a case of emergency, providing relevant data would be prioritised and a government official might find less difficulties. Nevertheless, the difficulties in reaching some professional categories remain a problem in case of an outbreak.

## Conclusion

Valuable insight into the travel patterns of Swedish professionals has emerged although the implications of the study largely concern the highly infectious diseases. The results of this study indicate that in Sweden a highly contagious disease might spread over a large area in the time span of one incubation period. The difficulties in reaching professionals visiting farms could be a problem in case of an outbreak.
